# Boosting teamwork between scrub nurses and neurosurgeons: exploring the value of a role-played hands-on, cadaver-free simulation and systematic review of the literature

**DOI:** 10.3389/fsurg.2024.1386887

**Published:** 2024-03-15

**Authors:** Camilla de Laurentis, David Pirillo, Andrea Di Cristofori, Alessandro Versace, Tommaso Calloni, Andrea Trezza, Valentina Villa, Lucia Alberti, Angelo Baldo, Federico Nicolosi, Giorgio Carrabba, Carlo Giussani

**Affiliations:** ^1^ School of Medicine and Surgery, Università Degli Studi di Milano Bicocca, Milan, Italy; ^2^ Neurosurgery, Fondazione IRCCS San Gerardo dei Tintori, Monza, Italy; ^3^ Neurosurgery, ASST Fatebenefratelli Sacco, Milan, Italy; ^4^ Operating Room, Fondazione IRCCS San Gerardo dei Tintori, Monza, Italy

**Keywords:** education, interprofessional, non-technical skills, operating room, roleplay, simulation, teamwork

## Abstract

**Background:**

Recently, non-technical skills (NTS) and teamwork in particular have been demonstrated to be essential in many jobs, in business as well as in medical specialties, including plastic, orthopedic, and general surgery. However, NTS and teamwork in neurosurgery have not yet been fully studied. We reviewed the relevant literature and designed a mock surgery to be used as a team-building activity specifically designed for scrub nurses and neurosurgeons.

**Methods:**

We conducted a systematic review by searching PubMed (Medline) and CINAHL, including relevant articles in English published until 15 July 2023. Then, we proposed a pilot study consisting of a single-session, hands-on, and cadaver-free activity, based on role play. Scrub nurses were administered the SPLINTS (Scrub Practitioners’ List of Intraoperative Non-Technical Skills) rating form as a self-evaluation at baseline and 20–30 days after the simulation. During the experiment, surgeons and scrub nurses role-played as each other, doing exercises including a simulated glioma resection surgery performed on an advanced model of a cerebral tumor (Tumor Box, UpSurgeOn®) under an exoscope. At the end, every participant completed an evaluation questionnaire.

**Results:**

A limited number of articles are available on the topic. This study reports one of the first neurosurgical team-building activities in the literature. All the participating scrub nurses and neurosurgeons positively evaluated the simulation developed on a roleplay. The use of a physical simulator seems an added value, as the tactile feedback given by the model further helps to understand the actual surgical job, more than only observing and assisting. The SPLINTS showed a statistically significant improvement not only in “Communication and Teamwork” (*p* = 0.048) but also in “Situation Awareness” (*p* = 0.031).

**Conclusion:**

Our study suggests that team-building activities may play a role in improving interprofessional teamwork and other NTS in neurosurgery.

## 1 Introduction

In recent years, non-technical skills (NTS) have been shown to play a pivotal role in every type of job. Not only should workers have and train in specific technical skills, they should also demonstrate and refine their interpersonal skills, such as efficient communication, leadership, and collaboration and teamwork, as well as their cognitive skills, represented by flexibility and coping attitudes, awareness of the situation, and decision-making (1–4). NTS can help in improving the quality and safety of work, on the one hand, and in allowing a reduction of stress, anxiety, and workload for the whole team, on the other hand—a beneficial effect on everyone's mental health.

One of the most important NTSs is teamwork, the ability to collaborate with colleagues toward a common aim, listening to them and helping if needed, allowing one to speed up processes while providing a high level of safety and also a calm, friendly environment for every team member.

NTS and teamwork, in particular, appear to be as important as technical skills (TS) to obtain high-quality results of jobs, both in the environment of business and companies (3, 4), in aviation (5), as well as in healthcare (6, 7). In surgical specialties, teamwork is mandatory, for example, in the operating room (OR) where errors could be fatal, and a collaborative environment is needed for the wellbeing of OR personnel. A safe and relaxed climate ultimately facilitates a smooth process and contributes to patient safety during the whole perioperative period (8, 9). Teamwork has been reported for example as a mainstay of general surgery, orthopedics, and plastic surgery (10–12), carrying positive consequences both on safety attitudes and results, and on the climate and personnel's wellbeing.

The theme of NTS in neurosurgery has not been explored much yet, and teamwork in neurosurgery is even more obscure in the literature. In particular, few publications have evaluated interprofessional teamwork in the neurosurgical OR and interventions intended as “team-building activities”.

The aims of our study were two: first, reviewing the literature about teamwork at the neurosurgical OR table; second, propose a simulation project as a team-building activity, specifically studied for scrub nurses working in neurosurgery and neurosurgeons.

## 2 Materials and methods

### 2.1 Systematic review

First, we conducted a systematic review of the existing literature on the topic “teamwork at the neurosurgical table” by searching the main medical and nursing databases, i.e., PubMed (Medline) and CINAHL, from the creation of the databases themselves until 15 July 2023. We included articles without restrictions about their publication status (fully published articles, online-ahead-of-print articles), in the English language, that could study the topic from surgeons’, trainees’, and scrub nurses’ points of view. Articles about perceptions or evaluation of the teamwork in the neurosurgical OR, or multi-specialty studies involving at least a neurosurgeon were eligible. Studies concerning interventions to improve teamwork were included when they dealt specifically with neurosurgery/neurosurgical procedures.

We excluded articles only describing multi-disciplinary surgeries (without evaluation of the value of teamwork), studies limited to other surgical domains or studying teamwork among anesthesiologists and neurosurgeons, publications about teamwork outside the OR (in the ward, in the whole healthcare system, in the rehabilitation process, and for emergent fire events). Publications that only mentioned scrub nurses working in “a wide range of specialties” or “in every specialty,” without further specification, were also excluded. We also excluded non-English articles, editorials, literature reviews, commentaries/perspectives/opinions, newspaper articles, proceedings or abstracts, and dissertations.

The systematic review was conducted and reported according to the Preferred Reporting Items for Systematic Reviews and Meta-Analyses (PRISMA) Statement 2020 (13) (Data Sheet 1 in the supplementary material).

We searched on PubMed for each of the following words: “teamwork”, “team building”, “team working”, and “non-technical skills”, combined with each of the following other words: “neurosurgery”, “surgery”, and “operating room”. We repeated the same procedure on CINAHL. We eliminated duplicates and then screened all titles and abstracts of the uniquely obtained articles. The publications assessed for eligibility were analyzed through reading of the full-texts and the relevant articles were finally included.

Afterward, we reviewed the references of the relevant studies as additional sources of eligible articles.

Data of the eligible works were obtained through careful analysis of full text by one author and checked by another. If a shared choice could not be reached between the two authors, a third surgeon was called to evaluate the most suitable solution.

### 2.2 Simulation scenario

**Figure 1:**
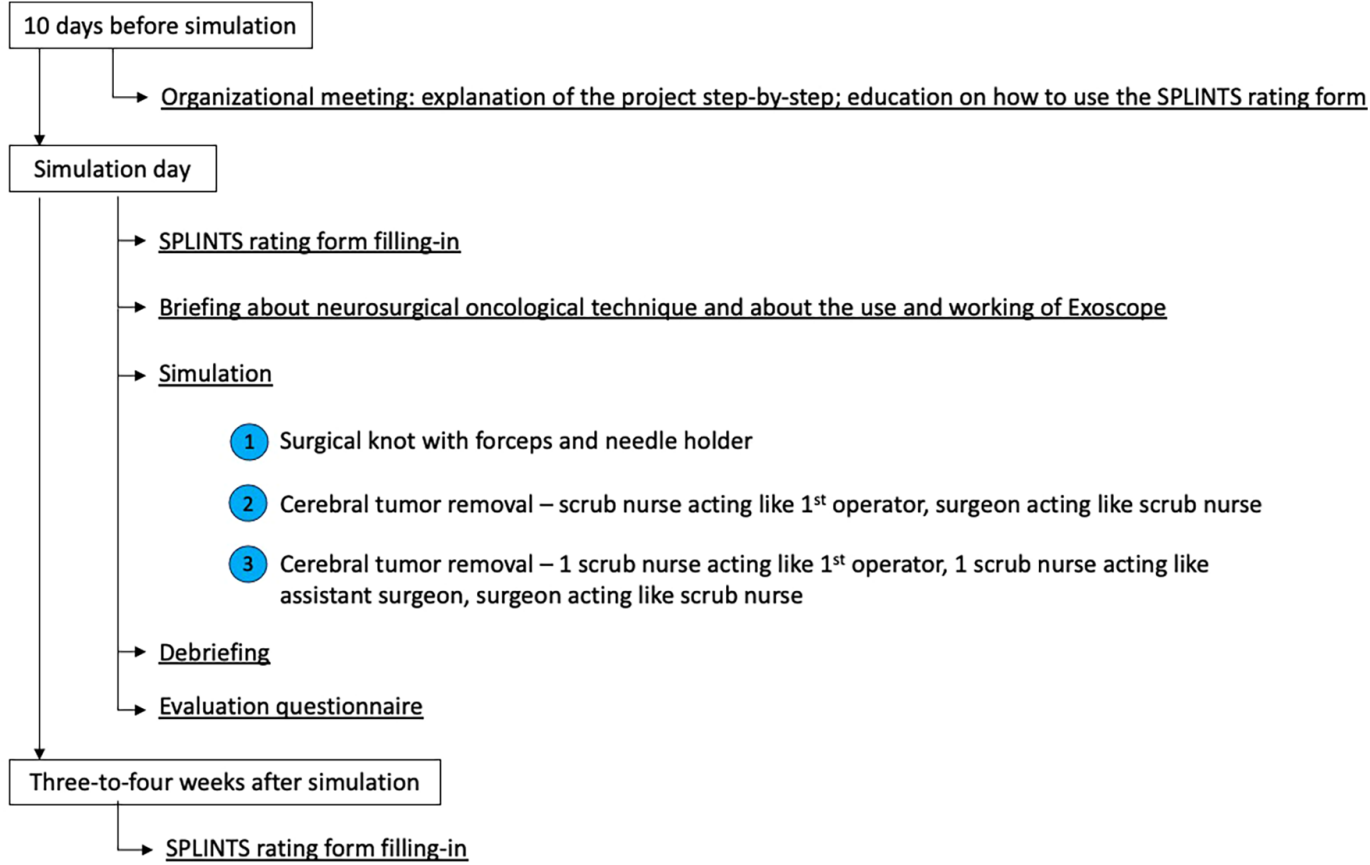
Flowchart schematically describing our simulation project step by step.

The second part of our article focuses on a pilot, explorative study carried out at our Institution (Fondazione IRCCS San Gerardo dei Tintori) to test the utility, appreciation, and value in boosting teamwork between scrub nurses and neurosurgeons of a simulation experience (Figure 1). This experience was a single-session, hands-on, and cadaver-free activity, based on role play, and it was specifically organized for scrub nurses working in neurosurgery (dedicated neurosurgical scrub nurses and scrub nurses taking part in emergent procedures including neurosurgical ones). An organizational meeting was held 10 days before the beginning of the simulation sessions, explaining the project step by step and how to use the self-evaluation questionnaire.

On the day of the simulation, first, scrub nurse participants were asked to complete the SPLINTS (Scrub Practitioners’ List of Intraoperative Non-Technical Skills) rating form as a self-evaluation at baseline (14–17). SPLINTS is a behavioral rating system, developed by psychologists and OR teams, that was originally designed to observe and rate scrub nurses in the OR. It comprises three main categories (situation awareness, communication and teamwork, and task management), each one further developed into three more specific subcategories, as explained in the User Manual (16). It is a rating system specifically developed for scrub nurses, in the same way as NOTECHS (18), ANTS (19), and NOTSS (20) were created for pilots, anesthesiologists, and surgeons, respectively.

**Figure 2:**
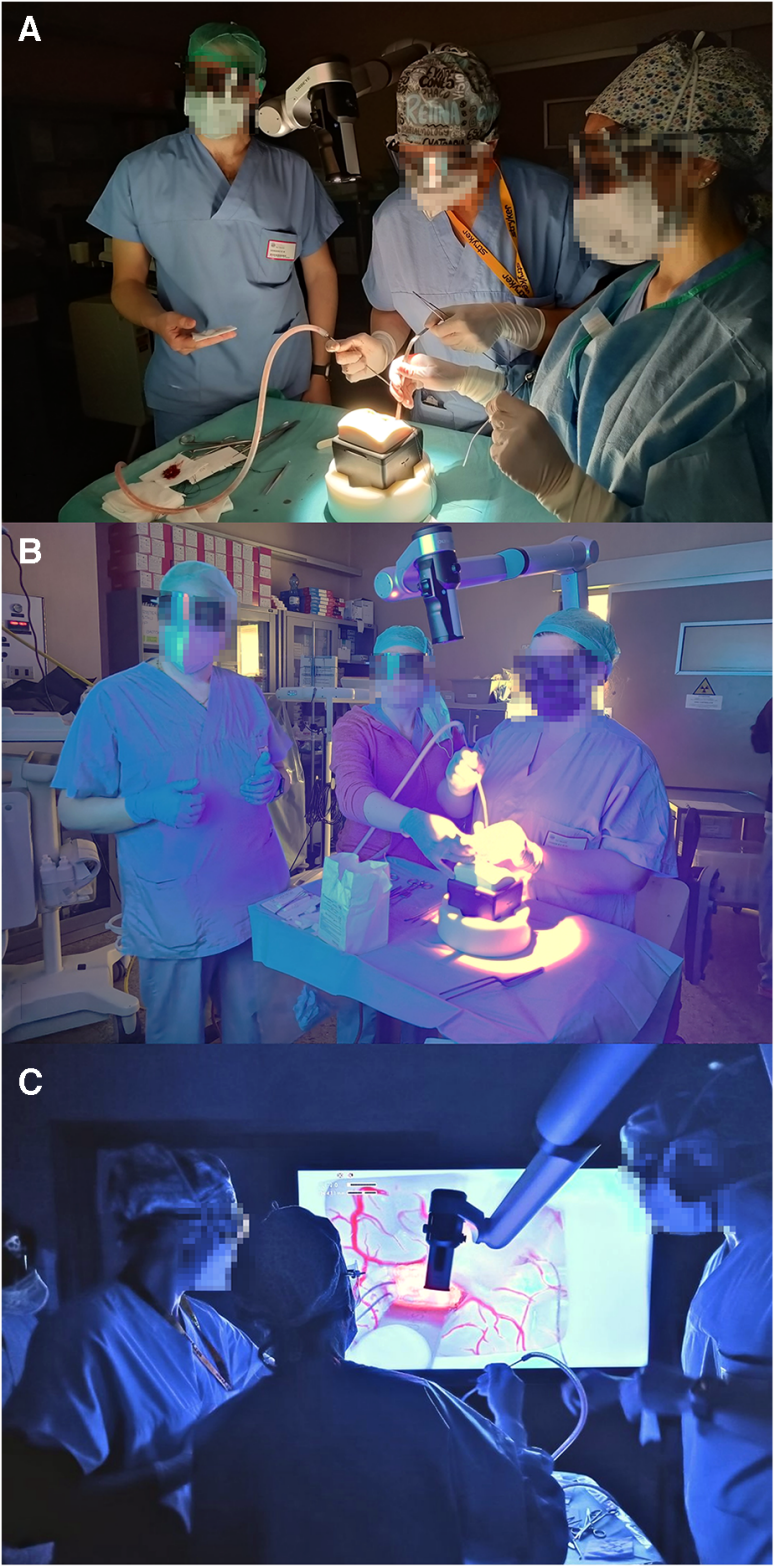
Pictures taken during some of the simulation sessions. (**A**) A scrub nurse is playing the first surgeon, a second scrub nurse is playing the assistant surgeon, and the neurosurgeon cares for the operating table. (**B**,**C**) Exercise made with the help of the blue filter for 5-ALA. In (**B**), the position of the participants in front of the exoscope screen is shown; in (**C**), all participants are seen looking at the exoscope screen.

A briefing about the neurosurgical oncological technique and the use and working of Exoscope was then offered to the participants. For the actual simulated surgery, a role switch was performed: the scrub nurse played the role of the surgeon, while the surgeon acted like a neurosurgical scrub nurse. Then, the session was developed into three exercises, executed with an exoscopic microsurgical technique. First, the scrub nurse had to make a surgical knot with forceps and a needle holder, looking at the monitor of the Exoscope. Second, they had to perform a cerebral tumor removal onto a model of intrinsic cerebral neoplasm (Tumor Box, UpSurgeOn®), with the support of the neurosurgeon who had prepared the surgical table and is now passing instruments. They also could try and understand the use of BLUE 400 filter for 5-aminolevulinic acid (5-ALA) vision, having the model a fluorescent tracer simulating 5-ALA. Third, the simulation was further developed with the support of a second scrub nurse impersonating the assistant surgeon (Figure 2). An observer noted every relevant comment or evaluation expressed during the whole session.

At the end of the simulation, a debriefing took place, underlining the acquired skills, stressing the understanding of the other's role, and giving the participants the chance to ask questions. Every participant—scrub nurses as well as neurosurgeons—was finally asked to complete an evaluation questionnaire with seven questions with a five-point Likert scale for answers, with the possibility to freely add comments. The questionnaire was adapted from a previous article by Shapiro (21) that had proposed a similar team-building experience in another domain of medicine.

The SPLINTS rating form was re-administered as a self-evaluation after three to four weeks after the experience. Every involved scrub nurse took part in at least three neurosurgical procedures in the intervening period, to better evaluate the practical value of the experience in improving their NTS in neurosurgery. The questionnaire results were collected anonymously. Supplementary Image 1 shows the evaluation questionnaire; the SPLINTS rating form can be found at the end of the user manual (16).

### 2.3 Statistical analysis

An online open-source software, jamovi® (www.jamovi.org) (22), was used for statistical analyses. The sample was described by means of the usual descriptive statistics. To compare independent discrete variables, we applied the Student's *t*-test. A threshold of *p* < 0.05 was set for statistical significance.

## 3 Results

### 3.1 Systematic review

As mentioned previously, we searched first on PubMed, and then on CINAHL. We obtained a total of 6,415 articles (4,145 from PubMed, 2,270 from CINAHL), of which 2,066 were duplicated. At this point, all titles and abstracts of the unique 4,349 articles were screened. A total of 292 studies were selected, but 47 of them were not retrievable. We finally assessed 245 articles for eligibility, analyzing full-texts, and excluded 211 of them for the following reasons: 88 because they specifically involved specialties other than neurosurgery; 51 because they did not specify involved surgical specialties; 57 because they were types of articles to be excluded; 15 treated irrelevant themes; and 3 were not in English.

From the references of the relevant studies, we further evaluated 11 eligible articles, and 3 were included.

**Figure 3:**
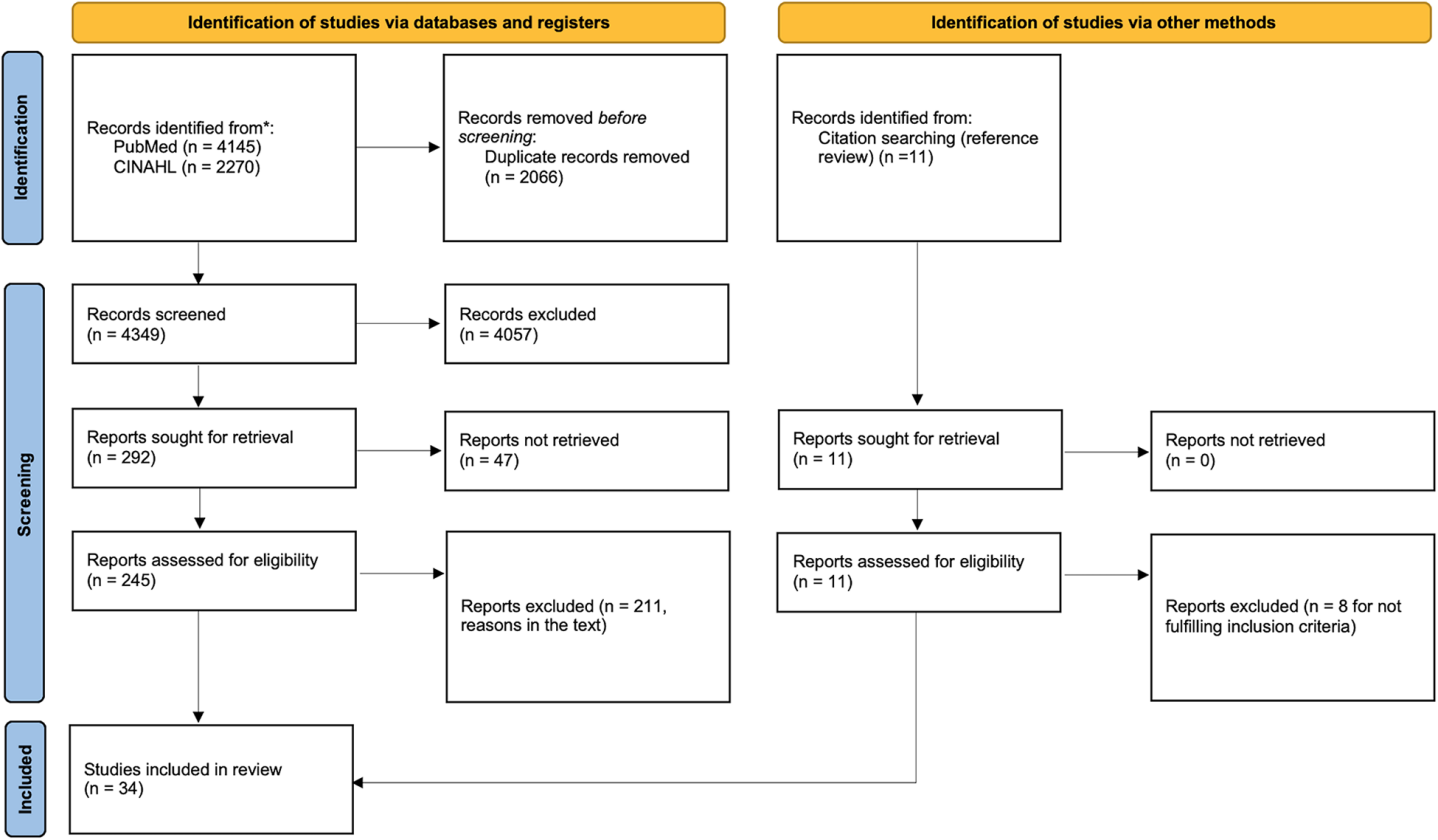
PRISMA 2020 flow diagram for new systematic reviews that included searches of databases, registers, and other sources. The flow chart of the inclusion process based on the “PRISMA 2020 flow diagram” [(see (13)].

The publications finally included in the study amounted to 34 (Figure 3).

We divided the included studies into two groups: the ones dealing with our theme exclusively in neurosurgery in Table 1 (23–36), and the ones studying teamwork and possible other NTS in many specialties, neurosurgery included, in Table 2 (8, 9, 37–54).

**Table 1:** Studies included in the systematic review, dealing with our theme exclusively in neurosurgery.

*N* ^a^	First author (reference)	Year	Teamwork among	Type (intervention/observation)	Method	Themes and findings
1	Hénaux (23)	2019	Scrub nurses and neurosurgeons	Observation	Audio and video recordings analyses	Familiarity; teamwork decreases workflow disruption
2	Lau (24)	2012	All neurosurgical OR^b^ members	Intervention	Creation of an educational safety video	Underlined in video is the importance of teamwork and communication for safety
3	Le Duff (25)	2023	Scrub nurses and other OR^b^ members	Intervention	"Virtual OR^b^ of Errors” scenario, craniotomy	VR^c^ training can improve NTSs^d^ (among which is teamwork)
4	Lepänluoma (26)	2013	Surgeons, anesthetists, circulating nurses	Intervention	Questionnaires pre- and post-introduction of checklist	Checklists improve safety-related performance, better communication, and teamwork
5	McLaughlin (27)	2014	All neurosurgical OR^b^ members	Observation	Questionnaire	Time-out process improves safety, not teamwork
6	Michinov (28)	2014	Neurosurgeons and other OR members	Observation	Development of behavioral marker system for assessing neurosurgical NTSs^d^	Scale is a structured approach to assessing NTSs^d^, teamwork also, in nsgy^f^
7	Pfandler (29)	2018	Surgeons, anesthetists, nurses	Observation	Interviews, observations, consensus on vertebroplasty	Define steps of vertebroplasty for all OR^b^ professions; guide for teamwork training
8	Pfandler (30)	2019	Spine surgeons and other OR^b^ members	Observation	Observational Teamwork Assessment for Surgery in simulated vertebroplasty environment	Higher level of tech skills associated with better NTS^d^, teamwork also, in spine surgeons
9	Sharp (31)	2005	Neurosurgeons	Observation	Review of surgical series	Mentoring new surgeons, selecting their cases to flatten LC^e^ (teamwork introduction)
10	Stevens (32)	2019	All neurosurgical OR^b^ members	Observation	Neurodynamic models from electroencephalography-derived measures	Similar electroencephalography traces in team members, reflecting teamwork
11	Zuckerman (33)	2012	OR nurses and other OR^b^ members	Intervention	Introduction of the Surgical Debrief Checklist	Invention to pilot application of a debriefing module to improve teamwork; safety
12	Ferroli (34)	2012	All neurosurgical OR^b^ members	Observation	Analysis of near misses through an aviation-derived prototype of incident reporting	Lack of teamwork is a contributing factor to near misses
13	Couat (35)	2013	All neurosurgical OR^b^ members	Observation	Direct observation, analysis of video of surgery	Briefing and checklists in OR^b^ improve teamwork and performance
14	Oszvald (36)	2012	All neurosurgical OR^b^ members	Observation	Review of surgical series	Pre-operative time-out synchronizes team members (improves teamwork), improves safety

^a^ *N*, number.

^b^ OR, operating room.

^c^ VR, virtual reality.

^d^ NTS, non-technical skills.

^e^ LC, learning curve.

^f^ nsgy, neurosurgery.

**Table 2:** Studies included in the systematic review, dealing with our theme in many specialties, neurosurgery included.

*N* ^a^	First author (reference)	Year	Teamwork among	Surgeries/units included, other than neurosurgery^c^	Type (intervention/observation)	Method	Themes and findings
15	Anderson (37)	2011	All OR^b^ members	Gen^c^	Observation	Social relationships analysis	Familiarity; nurses can be core members
16	Anton (38)	2021	Surgeons and others OR^b^ members	Cardiothor^c^	Observation	Questionnaire with STAI-6 and Surg-TLX; NOTSS^d^	Stress negatively affects NTS^e^; familiarity
17	Arad (8)	2022	All OR^b^ members	Gen, Ortho, Gyn, ENT, Uro, Plastic, Vasc, Cardio, Ophth^c^	Observation	Observations of surgical cases + interviews	Predicting teamwork and its lack; safety
18	Bogdanovic (39)	2015	All OR^b^ members	Plastic, Ortho, Gen^c^	Observation	Interview	Coordination means safe performance
19	Catchpole (40)	2010	All OR^b^ members	Maxillofacial, Vasc^c^	Intervention	Oxford NOTECHS^f^, SAQ^g^ and ethnographic observations post-intervention	Efficacy of aviation-style teamwork training
20	Cruz (31)	2019	All OR^b^ members	Cardiothor, Colorect, Gen, Gyn, Ophth, Ortho, ENT, Ped, Plastic, Transp, Uro, Vasc^c^	Observation	Questionnaire about pre-operative communication	Different perceptions of pre-operative communication among team members
21	Etherington (42)	2021	All OR^b^ members	Gen^c^, Uro, Ortho, Gyn, ENT, Onco, Plastic, Trauma^c^	Observation	Interview	Gender influence on teamwork
22	Etherington (43)	2021	All OR^b^ members	Gen^c^, Ortho, Uro, Gyn, ENT, Plastic, Thor, Trauma^c^	Observation	Interview	Barriers and enablers to teamwork
23	Eyigor (44)	2022	Residents and other OR^b^ members	Gyn, General, Ortho, ENT, Ophth, Plastic, Uro, Cardiovasc, Thor, Ped^c^	Observation	New scale (survey with 28 five-point Likert items)	Educational climate for residents
24	Finn (45)	2008	All OR^b^ members	Gen, Ortho, Spine, Vasc, ENT^c^	Observation	Ethnographic study (observation of surgical cases)	Teamwork produces unintended divisive effects
25	Gadjradj (46)	2019	Spine surgeons and other OR^b^ members	Ortho, Spine^c^	Observation	Questionnaire (SAQ^g^, expectations)	High scores in the teamwork domain of SAQ^g^; safety
26	Gillespie (9)	2013	All OR^b^ members	ENT, Vasc, Cardio, Gen, Ophth, Maxillofacial, Plastic, Uro, Ortho^c^	Observation	Ethnographic study (observation of surgical cases)	Teamwork and safety depend on communication; safety
27	Aukrust (47)	2021	Nurses in neurosurgery and colleagues	Nurses ICU, nurses neurosurgical unit and ped neurosurgical ward^c^	Intervention	Interviews post-intervention (training program in Norway)	After training, Malawian nurses gained NTS^e^, in particular teamwork
28	Kuy (48)	2016	All OR^b^ members	Gen, Vasc-thor, Ortho, Uro, Ophth, ENT, Podiatry^c^	Intervention	Crew resource management training; questionnaires post-intervention	Improvement on many points, including teamwork; safety
29	Leach (49)	2009	All OR^b^ members	Cardiovasc, Thor, Vasc, Ortho, Transp, Emerg, Uro, Head-neck, Gyn^c^	Observation	Direct observations and interviews	Different types of coordination in the team
30	Singer (50)	2015	All OR^b^ members	Gen, Ortho, Gyn/Uro, Cardiovasc, other^c^	Observation	Direct observations, rated with two new tools	Association surgical checklist performance-perceptions of OR^b^ teamwork; safety
31	Sonoda (51)	2017	Scrub + circulating nurses and other OR^b^ members	Gen, Ortho, Gyn, ENT, Uro^c^	Observation	Questionnaire	Most OR^b^ nurses perceive teamwork, but also stress
32	Su (52)	2022	Surgeons	Ortho^c^	Observation	Review of surgical series	With teamwork, learning curve of a new surgeon may be flattened
33	Urpo (53)	2021	Scrub + circulating + anesthesiology nurses and other OR^b^ members	Day surgery, Gen^c^, Ortho, Uro, other^c^	Observation	Questionnaire	OR^b^ nurses’ primary role and shift have connections to teamwork
34	Witmer (54)	2023	All OR^b^ members	Cardio, Gen^c^, Colorect, Uro, Ortho, Gyn, Thor, Transp, Vasc, Plastic, ENT, Trauma, other^c^	Observation	Terminals placed in the OR† (in-room survey system)	Satisfaction about teamwork depends on different variables

^a^ *N*, number.

^b^ OR, operating room.

^c^ Gen, general surgery; Cardiothor, cardiothoracic surgery; Ortho, orthopedic surgery; Gyn, obstetrics/gynecology; ENT, otorhinolaryngology; Uro, urology; Plastic, plastic surgery; Vasc, vascular surgery; Cardio, cardiologic surgery; Ophth, ophthalmology; Maxillofacial, maxillofacial surgery; Coloret, colorectal surgery; Ped, pediatric surgery; Transp, transplant surgery; Onco, oncological surgery; Trauma, trauma surgery; Cardiovasc, Cardiovascular surgery; Thor, thoracic surgery; Spine, spine surgery; ICU, intensive care unit; Vasc-thor, vascular and thoracic surgery; Emerg, emergency surgery; Head-neck, head-neck department surgery.

^d^ STAI-6, state-trait anxiety inventory six-item version; Surg-TLX, surgery task load index.

^e^ NTS, non-technical skills.

^f^ NOTECHS, operating theater team non-technical skills system (assessment tool).

^g^ SAQ, safety attitudes questionnaire.

Table 1 shows that only one studied the specific relationship among scrub nurses and neurosurgeons at the table (23), while the others globally looked at teamwork in the whole neurosurgical OR environment (24, 26, 27, 29, 32, 34–36), focusing on neurosurgeons (28, 30, 31), or studied exclusively nurses’ tasks or points of view (25, 33).

Similarly, only 4 out of 14 studies tried an intervention to improve teamwork (24–26, 33). The interventions included (1) the production of a video, (2) a virtual reality simulation in which nurses had to find errors in the preparation of a craniotomy, and (3) and (4) the introduction of a checklist.

Very interestingly, the work by McLaughlin et al. concluded that the time-out process may improve safety, but not teamwork—the only “negative” report about teamwork in this group of articles (27).

In Table 2, it is evident that similarly to exclusively neurosurgical reports, the majority of publications (13 out of 20) globally looked at teamwork among the whole staff; 3 focused primarily on surgeons (38, 46, 52), 3 on nurses (47, 51, 53), and 1 on residents (44). The interventional activities are also limited: 3/20, including aviation-style teamwork training (40), a training program in Norway for Malawian nurses (47), and crew resource management training (48).

In all the articles, the theme of safety is often stressed: teamwork and NTS, in general, are considered above all as tools to improve safety in the OR.

### 3.2 Population and SPLINTS rating form

A total of 10 scrub nurses and 5 neurosurgeons participated in the simulation experience. Six of the nurses worked exclusively in neurosurgery, four were part of the emergency staff and were involved also in emergent neurosurgical cases. Nine were women and one, man. Every nurse had at least 2 years of experience in the OR. The five neurosurgeons included four attending surgeons and one final-year resident.

The results of the comparison between the pre- and post-experience self-evaluations are provided in Table 3. Very interestingly, the rating scrub nurses gave themselves in the macro-categories “Situation Awareness” and “Communication and Teamwork” showed a statistically significant improvement from the pre- to the post-evaluation. The overall score also improved significantly (A in Table 3).

**Table 3:** Comparison between the pre- and post-experience self-evaluations.

A	
Question	*p-*value
Category 1	**0**.**031**
Category 2	**0**.**048**
Category 3	0.171
Overall score	**0**.**026**
B	
Category	*p*-value
1.1	0.067
1.2	**0**.**005**
1.3	**0**.**009**
2.1	0.235
2.2	**0**.**028**
2.3	0.196
3.1	0.111
3.2	0.087
3.3	0.153

Part A shows the *p*-value for the macro-categories. Part B shows the *p*-value for each item. Bold values are statistically significant (*p*-value set at <0.05).

When looking at the rating scrub nurses wrote in every single item, three showed statistically significant improvement (“Recognizing and understanding information”, “Anticipating”, “Exchanging information”) and all showed improvement tending toward statistical significance (B in Table 3).

### 3.3 Evaluation questionnaire

Among the items in the evaluation questionnaire, the last four questions specifically concerned teamwork training [4. The quality of the final debriefing for my understanding of the surgeon's work was high, 5. The experience was useful to understand the surgeon's point of view, 6. The experience helped improve my teamwork skills, 7. I would redo the activity to further improve my teamwork skills].

**Figure 4:**
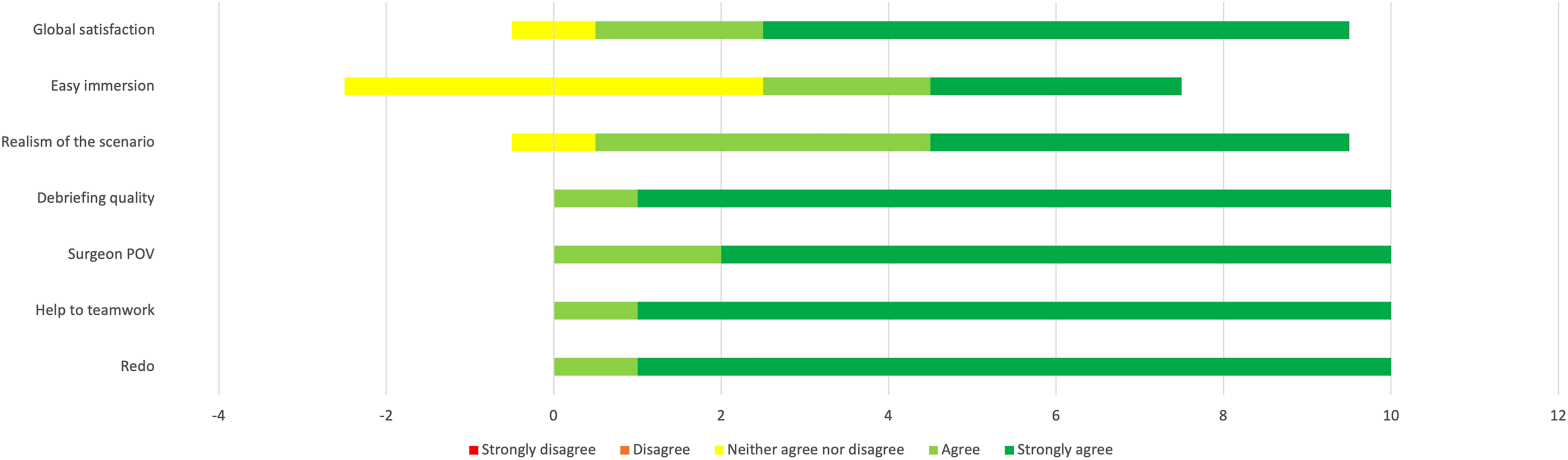
Results of the evaluation questionnaire administered to scrub nurses (*n* = 10). Answers are expressed on a five-point Likert scale.

**Figure 5:**
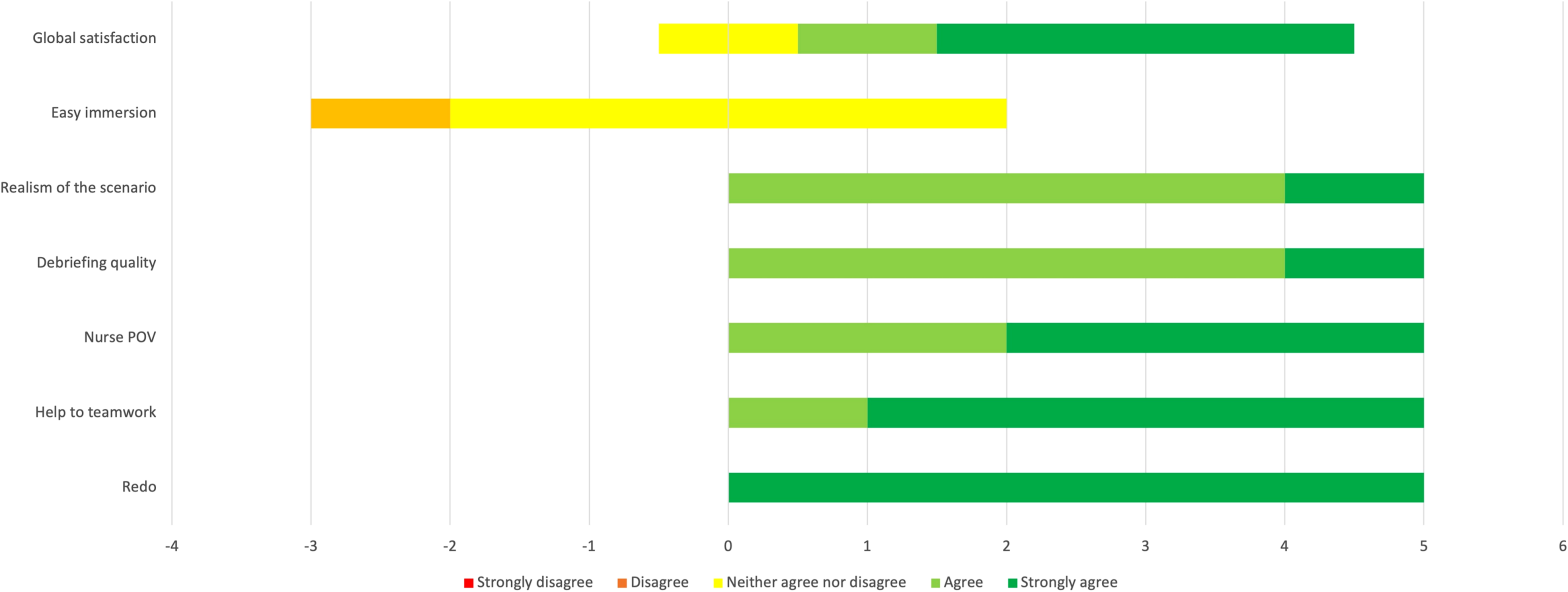
Results of the evaluation questionnaire administered to neurosurgeons (*n* = 5). Answers are expressed on a five-point Likert scale.

The results of the questionnaire administered to scrub nurses are shown in Figure 4, whereas the surgeons’ evaluations are represented in Figure 5.

As shown by the graphs, the results of the four items about teamwork were all positive in both groups (agree and strongly agree). The nurses scored items 5, 6, and 7 even higher than the surgeons. The surgeons were globally not satisfied by the ease of immersion in the simulation (four neurosurgeons answered “neutral”; one answered “disagree”), whereas half of the scrub nurses were satisfied to some extent (five agreed or strongly agreed with the sentence).

### 3.4 Free comments and observations

Every relevant comment orally expressed by participants during the sessions and noted by an external observer was qualitatively analyzed, in the same way as every possible note written at the end of the final evaluation questionnaire (Table 4).

**Table 4:** Free comments and observations from the evaluation questionnaire.

Repeat and improve	
Desire to repeat the experience	5
Try other boxes	1
Extend to other specialties	1
Improve realism of the whole scenario	1
Repeat with all the surgeons	1
Pleasure	
Pleasure of participating in such a simulation	2
Comments on surgeons’ work	
Difficulty in using exoscope	1
Many things to think about	1
Mocking	10

We grouped them into three main themes. The first group of comments was about repeating and improving the simulation experience. As already evident from the Likert items, five scrub nurses further underlined their desire to repeat the experience. Moreover, one suggested trying other boxes from UpSurgeOn® and exercises within neurosurgery, and another one mentioned the possible value of extending such an experience to other specialties. One nurse asked for a possible improvement in the realism of the global scenario and another suggested repeating the experience with all the surgeons, in particular the ones perceived as stricter and/or shyer, to try and strengthen a friendly and trusting relationship.

The second theme in free comments regarded the pleasure of participating in such a simulation, which was stressed by two participants. They felt they had agency and enjoyed the chance to ask questions, understand, and learn in a “safe,” tension-free setting.

The third group of comments was about the surgeons’ work. Two other nurses in particular commented on it: “I now understand how difficult it is to use the Exoscope”; “How many things do you surgeons have to think about, while operating!” Moreover, very interestingly, during each session, every single participant was light-heartedly mocking the other group's typical expressions and comments. For example, every nurse playing the surgeon exaggeratingly complained about the blockage of the aspirator, a typical complaint of the surgeon during everyday surgical cases.

## 4 Discussion

NTS are becoming more and more relevant in every type of job. Any type of worker should be able not only to acquire specific technical abilities, but also work on their interpersonal skills, such as leadership, efficient communication, and collaboration and teamwork, as well as their cognitive abilities, represented by awareness of the situation, decision-making, and flexibility and coping attitudes (1–4). The importance of NTS depends on their demonstrated role in both contributing to the improvement of quality and safety of work and in allowing the construction of a calm, friendly work environment for the whole team—which is beneficial to everyone's mental health and to the safety in the workflow.

Teamwork is a fundamental NTS, and it is in fact the ability to focus as a team on a common aim, listening to colleagues and offering them help. Teamwork contributes to speeding up processes and workflows while maintaining a high level of safety at each step and also guaranteeing a healthier environment for team members.

Our literature review showed a limited number of articles about interprofessional teamwork among neurosurgeons and scrub nurses in the OR, mainly focusing on observing the existing situation (Tables 1, 2). Interestingly, the vast majority of articles did not focus on the specific relationship between scrub nurses and neurosurgeons but globally looked at teamwork among the whole staff, *exclusively* in neurosurgery or *also* in neurosurgery. We strongly believe that this relationship should be studied and taken care of on its own because it requires a set of shared skills, know-how, and knowledge that is never required in the whole OR environment. Another point that is worth stressing is that few interventions have been proposed to try and improve NTS and teamwork in particular (7 out of 34, 20.6%), while as mentioned the others generally reported measurements and observations about the *status quo* of teamwork in the OR. When analyzing these interventions, we can find three passive approaches—production of a video in one case (24), introduction of a checklist in two cases (26, 33)—and four active ones—a virtual reality simulation for nurses (25), a nurse-centered training program in a foreign country (47), and two training models borrowed from aviation (40) and crew resource management (48).

It seems clear that no activity, neither active nor passive, can be found in the literature reproducing the exclusive, real-life relationship among scrub nurses and neurosurgeons in the OR, and with the ultimate aim of trying to improve the quality of that working relationship.

Thus, we developed the project of our hands-on, cadaver-free simulation, which may therefore be considered one of the first experiences in the literature describing a neurosurgical team-building activity to boost interprofessional teamwork in the OR, specifically addressing scrub nurses.

Although missing in neurosurgery, a certain number of similar activities has already been proposed in other surgical specialties, for example, plastic surgery (12), emergency medicine (55), and gynecology (56), often based on concepts taken from aviation (5, 34, 40). The initial experiences of NTS in healthcare historically came from aviation, in which pilots are required not only to perfectly demonstrate their technical abilities but also to cultivate their interprofessional skills and cognitive skills to provide safety during their job activity. Our review reports some recalls to aviation (34, 40).

We believe it is important to underline that beneficial effects on teamwork could be seen on safety but also on the work environment (8). In fact, based on our review, the role of teamwork in a professional’s wellbeing appears to be quite overlooked. To us, this seems like a big omission. Teamwork is surely pivotal for safety in the OR, but safety for the patient improves through the reduction of stress, anxiety, and workload for the team members. Moreover, mental health is a right of every worker. Therefore, a friendly, familiar, and harmonious work environment surely gives benefits to everyone—the care team and patients.

Our simulation may suggest the role of team-building activities in teamwork improvement in neurosurgery (Table 3). For the sake of clarity, the reasons for choosing the SPLINTS rating form (14, 15, 17) were multiple and are now discussed. First, it is specifically aimed at scrub nurses, which was our primary criterion. Second, it is user-friendly and easy to understand, and we found it to be both very precise and non-redundant and thus efficient. A goal for us during the design of this study was not to hinder the professional activity or personal life of study participants by limiting the amount of time needed to take part. Third, as specified in the manual (16), although it was designed for evaluation by an external observer, it can also be used for self-evaluation. Finally, it also allows a global evaluation not only of teamwork but also of other NTS, giving clues about a more comprehensive value of our project.

The SPLINTS rating form items showed a statistically significant improvement from the pre- to the post-evaluation not only in the macro-category “Communication and Teamwork” but also in another NTS that is “Situation Awareness,” while the results were not significant for “Task management.” This seems straightforward, as the first two categories imply putting oneself in somebody else's shoes, that is exactly what is experienced in the role switch of our simulation. On the contrary, the proposed activity does not focus on the management of different tasks.

When looking at the single items, they tend to have statistical significance although this is less evident than in the macro-categories. We think this may be related to multiple factors. First, dividing single specific behaviors within a global category may be difficult for people not having a long, focused training on NTS evaluation. Second, the evaluation scale was a four-point one, which may contribute together with the number of participants in making the otherwise relevant chances not cross the statistical significance threshold. Third, self-evaluation also includes judgments about oneself and the mood of the day, being therefore more variable than an external evaluation.

All the participants, neurosurgeons as well as scrub nurses, globally rated the experience positively (Figures 4, 5). Scrub nurses in particular showed a high level of enthusiasm, suggesting repeating the experience and in some cases even opening it to other specialties. They felt more active than usual and perceived a tension-free environment in which learning was facilitated. In particular, they demonstrated a particular utility in understanding the neurosurgeon's point of view, difficulties, and challenges. The use of a physical simulator such as the Tumor Box from UpSurgeOn® seems an added value, as the tactile feedback given by the model further helps understand the actual surgical job, more than only observing and assisting.

Unexpectedly, in all sessions every participant mocked the other profession's typical expressions and comments, often causing a general laugh. This may suggest a role of team-building activities also in exorcizing their own fears and awe, in facing possible resentments and disagreements, and in helping the other professionals resize their exaggerated behaviors.

The realism of the situation and the general context, specifically in tasks related to preparation for surgery, patient positioning, and space management in the OR, was a less appreciated point, together with the ease of immersion in the simulation. In fact, the simulators used were designed to replicate the surgical field itself rather than the surrounding context (the surgical drapes, the patient, the spatial organization of the OR), which certainly diminished the perception of realism in terms of “faithful reproduction of all steps of a surgical session,” from patient arrival in the OR to drapes removal. This aspect could be further developed in future sessions. Moreover, it was suggested to repeat the simulation with all the neurosurgeons of the department, in particular those perceived as stricter and/or shyer ones, to try and strengthen a friendly and trusting relationship that could carry over to real—and often very challenging and stressful—scenarios.

Having a look at the whole of our results, we could imagine some possible practical research implications and evolutions of our pilot experience. With the introduction of cadaver-free simulation models, it is possible to assume that all dedicated neurosurgical scrub nurses may have the chance to simulate the role of the neurosurgeon in a safe scenario and to repeat these simulation sessions as needed, ideally arriving at the realization of a specific educational program. This may lead to a global reinforcement of all NTSs, in particular,

- increased perception of the neurosurgeon's needs in case of adverse intraoperative events (e.g., massive sudden bleedings);
- better step-by-step anticipation of the neurosurgeons’ needs, to speed up the surgical procedure and optimize the use of the operatory room time for each procedure;
- better help the neurosurgeon in surgeries performed in a single-surgeon setting, to make surgery more efficient;
- increase in awareness during surgery of complex cases, so as to have all the dedicated instruments available as soon as possible during surgery.

Furthermore, the establishment of such cadaver-free NTS programs for scrub nurses may be proposed for every specific setting. This might lead to the definition of specialized scrub nurses for each surgical specialty. Such an implementation of educational training of scrub nurses might be considered an evolution of the professional competencies of nurses as it happens in medical specialties or sub-specialties.

Our study shows some limitations, as already mentioned, and may suggest some future directions. First, the number of participants was extremely limited, given the voluntary recruitment and the nature of our research itself as a “pilot study.” This point limits, in fact, the impact of the statistical analysis and the generalizability of our findings. Ideally, the simulation should be repeated with as many scrub nurses working in neurosurgery as possible, and with all the neurosurgeons of the department, if not even in a multi-centric fashion. Second, self-evaluation allows a rapid, realistic evaluation of oneself, but includes one's own self-esteem, which is incredibly variable among people and even on different days. Thus, a similar session with a specifically trained external observation may overcome this limitation. Lastly, we did not evaluate the persistence of the improvements. In other words, we do not know if improvement in teamwork and situation awareness remains months after the simulation. Another evaluation could be administered at a certain time distance, as well as new “recall sessions” could be proposed. A pre-simulation theoretical meeting about teamwork and NTS could also be offered.

## 5 Conclusion

Our study represents one of the first experiences of a hands-on, cadaver-free neurosurgical simulation to boost teamwork in the neurosurgical OR. It was globally very well welcomed for its subjectively perceived utility. The present work may suggest that team-building activities could play a role in improving teamwork abilities and other NTS in neurosurgery. Future studies and sessions may support our findings and perhaps even further improve the efficacy of such interventions, also studying the possible sustainment of improvement.

## Data Availability

The original contributions presented in the study are included in the article/Supplementary Material, further inquiries can be directed to the corresponding author.
