# Supplementary figures and images for: Boosting teamwork between scrub nurses and neurosurgeons: exploring the value of a role-played hands-on, cadaver-free simulation and systematic review of the literature
Source: Front Surg. 2024 Mar 15;11:1386887. doi: 10.3389/fsurg.2024.1386887 (PMC10978771; doi:10.3389/fsurg.2024.1386887)

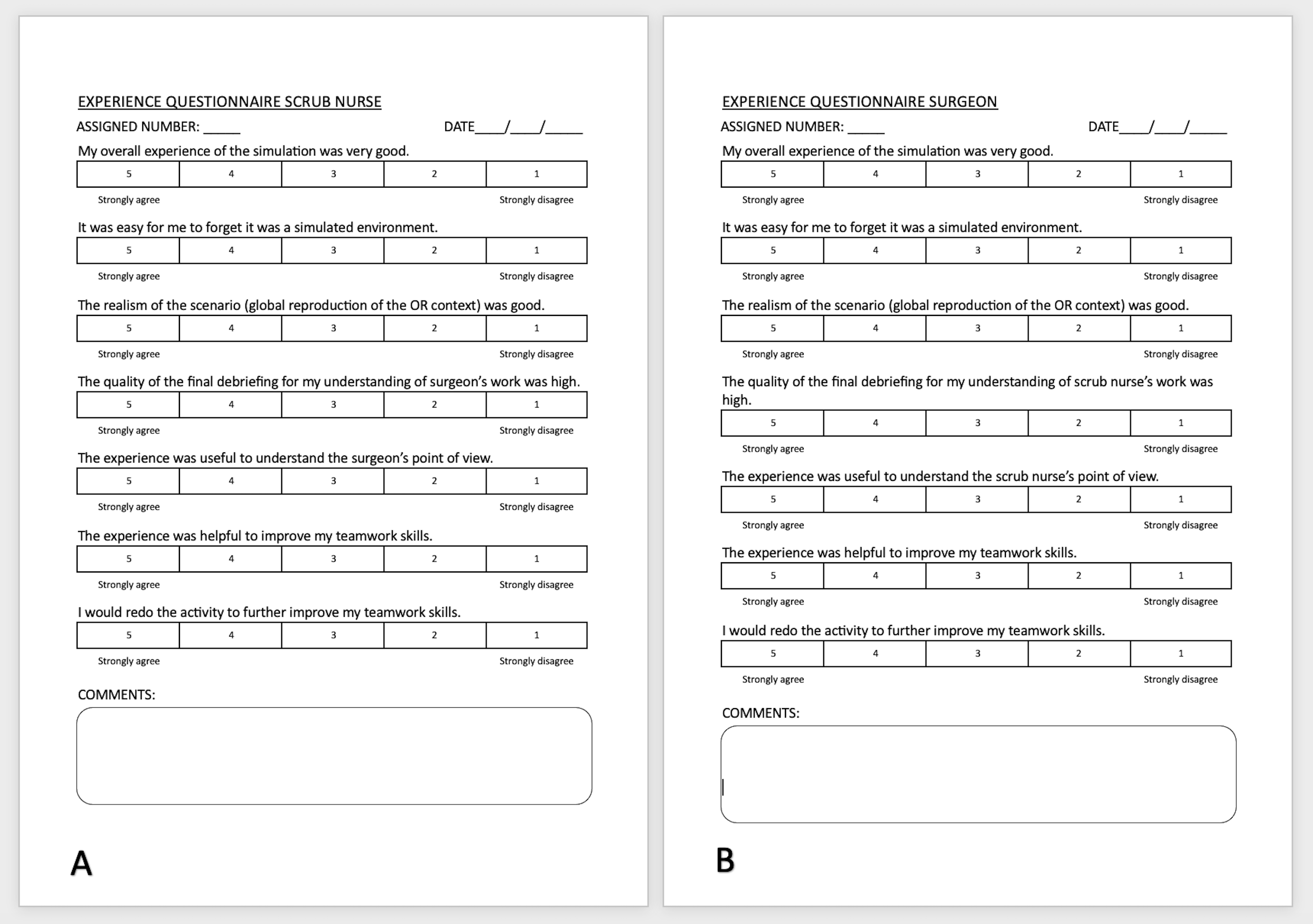

Supplement: Supplementary file 2 [file Image1.tiff]
